# Trends of inequalities in care seeking behavior for under-five children with suspected pneumonia in Ethiopia: evidence from Ethiopia demographic and health surveys (2005–2016)

**DOI:** 10.1186/s12889-021-10232-x

**Published:** 2021-02-01

**Authors:** Gebretsadik Shibre, Betregiorgis Zegeye, Dina Idriss-Wheeler, Sanni Yaya

**Affiliations:** 1 Department of Reproductive, Family and Population Health, School of Public Health, Addis Ababa University, Addis Ababa, Ethiopia; 2 HaSET Maternal and Child Health Research Program, Shewarobit Field Office, Shewarobit, Ethiopia; 3 Interdisciplinary School of Health Sciences, University of Ottawa, Ottawa, Canada; 4 School of International Development and Global Studies, University of Ottawa, Ottawa, Canada; 5 The George Institute for Global Health, Imperial College London, London, UK

**Keywords:** Pneumonia, Inequality, Care seeking behavior, Ethiopia, DHS, Children, Global health

## Abstract

**Background:**

Pneumonia is a leading public health problem in under-five children worldwide and particularly in Africa. Unfortunately, progress in reducing pneumonia related mortality has been slow. The number of children with symptoms of pneumonia taken to health facilities for treatment is low in Ethiopia, and disparities among sub-groups regarding health seeking behavior for pneumonia have not been well explored in the region. This study assessed the trends of inequalities in care seeking behavior for children under five years of age with suspected pneumonia in Ethiopia.

**Methods:**

Using cross-sectional data from the 2005, 2011 and 2016 Ethiopia Demographic and Health Surveys (DHS) and the World Health Organization’s (WHO) Health Equity Assessment Toolkit (HEAT), this study investigated the inequalities in health seeking behavior for children with suspected pneumonia. Four measures of inequality were calculated: Difference, Ratio, Slope Index of Inequality and Relative Index of Inequality. Results were disaggregated by wealth, education, residence, and sex with computed 95% Uncertainty Intervals for each point estimate to determine significance.

**Results:**

The percentage of under-five children with symptoms of pneumonia who were taken to a health facility was significantly lower for children in the poorest families, 15.48% (95% UI; 9.77, 23.64) as compared to children in the richest families, 61.72% (95% UI; 45.06, 76.02) in 2011. Substantial absolute (SII = 35.61; 95% UI: 25.31, 45.92) and relative (RII = 4.04%; 95% UI: 2.25, 5.84) economic inequalities were also observed. Both educational and geographic inequalities were observed; (RII = 2.07; 95% UI: 1.08, 3.06) and (D = 28.26; 95% UI: 7.14, 49.37), respectively. Economic inequality decreased from 2011 to 2016. There was no statistically significant difference between male and female under-five children with pneumonia symptoms taken to health facility, in all the studied years.

**Conclusions:**

Health care seeking behavior for children with pneumonia was lower among the poorest and non-educated families as well as children in rural regions. Policies and strategies need to target subpopulations lagging behind in seeking care for pneumonia treatment as it impedes achievement of key UN sustainable development goals (SDGs).

## Background

Pneumonia kills close to one million children under the age of five annually, representing 15% of under-five child mortality [1]. Although pneumonia affects children and families across the globe, South Asia and sub-Saharan Africa carry the heavy burden of this infectious disease [1]. In 2016, lower respiratory tract infections claimed more deaths than malaria and tuberculosis combined in the African continent [2]. Furthermore, pneumonia remains the leading cause of morbidity for young children in low-and-middle-income countries (LMICs) [3]. A recent meta-analysis revealed that the burden of pneumonia in East Africa ranged between 24 to 44% among children under five years of age [4]. Ethiopia is among the five countries globally (the other four being India, Nigeria, Pakistan, the Democratic Republic of Congo) which had more than half of all 2017 deaths resulting from childhood pneumonia [5]. Although reliable evidence is lacking in the country, it is estimated that one in 14 children in Ethiopia suffered from acute lower respiratory tract infection, mainly pneumonia [6].

Multiple risk factors are thought to cause pneumonia. While different microorganisms are implicated in the cause, several other underlying factors have been shown to play a significant role in facilitating the advancement of the disease. Malnutrition, HIV infection or exposure, and tobacco smoke exposure have been reported as important modifiable risk factors for pneumonia [3]. One of the leading risk factors for mortality due to lower respiratory infection among children under five is childhood wasting - it accounted for more than 61% of 2016’s lower respiratory deaths [7]. Increasing effective interventions that improve wasting, household air pollution, ambient particulate matter pollution, and expanded antibiotics may possibly reduce the burden of pneumonia [7].

Pneumonia caused by bacteria can be treated with antibiotics, but only one third of children with pneumonia, globally, receive the antibiotics they need [1]. In Ethiopia, only three in ten children with pneumonia receive treatment [6], suggesting a large gap between diagnosis and treatment. To mitigate costs of deaths and mortalities caused by pneumonia, scale up implementation of effective interventions are necessary for all children with symptoms of the disease [8]. The *Global Action Plan for Pneumonia and Diarrhea (GAPPD),* issued by the WHO and UNICEF, listed a set of interventions known to decrease deaths from these major killers, aiding countries in moving towards internationally agreed upon targets (SDGs) for child health [8, 9]. These include exclusive breast feeding, adequate complementary feeding and continued breastfeeding, vitamin A supplementation, immunization, reduced household air pollution as well as improved care seeking and referral [8]. However, few children receive these interventions [8], impeding efforts to attain the 2025 global goal of (i) reducing pneumonia related mortality to fewer than 3 per 1000 live births among children under five, (ii) 90% access to appropriate pneumonia case management and (iii) 80% coverage in every district of the country [9].

Pneumonia is often recognized as *“the ultimate disease of poverty”* [10]. The disproportionately higher concentration of the disease in low-income countries emphasizes the correlation between a countries’ income and child mortality from pneumonia [5]. LMICs carry 90% of the burden of pneumonia globally [8]. Within these poor countries, there also exists a substantial differential of pneumonia burden among the different population subgroups. Children born to the poorest families are more likely to suffer pneumonia than children born to more affluent families [8]. Large variations in the burden of pneumonia also exist across other dimensions of inequality including place of residence, wealth and maternal education [6].

The three-tier Ethiopian health care system provides health care services at the primary, secondary and tertiary levels. At the primary level, general and non-specialized health services are provided for communities at the district level. Since health services at this lowest point of contact are widely available in the country, poor individuals are likely to afford and access them for treatment of common childhood illnesses (i.e. pneumonia) and they remain an important component of the primary health care service [11, 12]. The flagship of the Ethiopian health care delivery system - the Health Extension Programme (HEP) - provides a wide-range of preventive and curative maternal, newborn and child health care to the rural community. The HEP provides integrated community case management (ICCM), which includes treatment of pneumonia in under-five children [13]. By bringing the health care services to the place where people reside, the HEP considerably cuts the transport and other related costs [13]. The secondary and tiery levels include general and specialized hospitals and cover large portions of the population.

To our knowledge, assessment of the inequality in health care seeking behavior for under-five children with suspected pneumonia in Ethiopia with respect to place of residence, sex, and socioeconomic status has not been done. In this paper, we approached inequality both at a point in time and over time to investigate patterns of disparity and suggest mitigating solutions. We used a scientifically sound and rigorous inequality analysis approach by the WHO. Findings can facilitate implementation of equity-oriented interventions to reduce observed disparity and preventable deaths from pneumonia, as set forth by the WHO/UNICEF integrated Global Action Plan for Pneumonia and Diarrhea (GAPPD) [9].

## Methods

### Brief overview of the study setting

As of 2020, Ethiopia has 114,964,588 people, and is the second most populous country in Africa [14]. Despite economic growth and a decrease in poverty over the past decade [15], with an average per capita 2017 Gross Domestic Product (GDP) of US$830, Ethiopia continues to be one of the poorest countries in the world [16]. Furthermore, Ethiopia sits in the bottom 10% when ranked by the Human Development Index (HDI), suggesting poor performance according to indicators of life expectancy, literacy, education, and a decent standard of living [17].

### Data sources

The offline version of the WHO HEAT software was used as both the source of data and tool for analysis for the study; the software has been described in detail elsewhere [18, 19]. The HEAT software provides a tool to complete descriptive analyses using both absolute and relative measures and produce key dimensions of inequality, allowing investigators to determine inequities within and between countries [20]. The data is drawn from the WHO’s Health Equity Monitor database which is derived from both the demographic and health surveys and the multiple indicator cluster surveys of more than 30 LMICs, including Ethiopia [20]. The software presents inequality analysis of more than 15 different summary measures such as education, residence, subnational regions, and sex. We used cross-sectional data from the 2005, 2011 and 2016 Ethiopia Demographic and Health Surveys (EDHSs) available in the HEAT software. The 2000 EDHS was not used in this study since it was not available in the software. The household surveys used a two-stage cluster design to draw samples. The territory of Ethiopia was stratified into various strata for the purpose of sampling, and enumeration areas (i.e., the primary sampling units [PSU] or clusters) were then selected through Probability Proportional to Size (PPS) in the first stage from each stratum [6]. In the second stage, 28 to 30 households were selected from within each PSU independently. The nationally representative DHS surveys collect information including topics such as anthropometric, demographic, socioeconomic, maternal health services, childhood illness, family planning and domestic violence. Women aged 15 to 49 years were the major source of data for the surveys, although data were also collected from under five children and men aged 15 to 59 years. More details on the methodologies of the respective surveys are available elsewhere [6, 21, 22].

### Selection of variables

Care seeking behavior for under-five children with suspected pneumonia was the primary variable for which inequality was measured. Four equity stratifiers were selected: economic status, maternal education, place of residence, and sex. DHS uses wealth index as a proxy for economic status which is classified by poorest, poor, middle, rich and richest. The wealth index is constructed in DHS using household assets and ownership through principal component analysis (PCA) as described in detail here [23] and deemed comparable across all the survey years. Maternal educational status was separated into no-education, primary education, and secondary education or higher, while place of residence was urban or rural.

### Data analysis

The latest version of the WHO’s HEAT software was used for analysis [18]. Datasets were analyzed and disaggregated by the four equity stratifiers: economic status, education, place of residence and sex. They were presented using the four summary measures of health inequality - Difference (D), Ratio (R), Slope Index of Inequality (SII) and Relative Index of Inequality (RII) - chosen from the pool of 15 measures available in the software [18]. These measures were chosen because they have wider application in health care inequality studies [24]. As recommended by the WHO, for equity studies, both simple and complex summary measures were calculated for each equity stratifier to better understand the inequality involved in health seeking behavior (by caregivers) for suspected child pneumonia - referred to hereon in as *health seeking behaviour for childhood pneumonia* [24]. Where relevant, we calculated complex measures for some of the dimensions of inequality to present the inequality from both complex and simple perspectives. The practice of adopting simple/complex and relative/absolute measures of inequality is necessary to produce evidence useful for decision makers and planners [24].

More details on the definitions, interpretations and calculation of summary measures are available elsewhere [18, 24]. Briefly, the Difference and Ratio are simple measures of health inequality, whereas SII and RII are complex measures. Also, while Difference and SII are absolute measures, RII and Ratio are relative measures. Simple measures work for pairwise comparison of a health indicator, but they do not consider subpopulation for an indicator with more than two categories (i.e. wealth index, sub-national regions). Complex measure estimates consider all the categories within an indicator.

Difference, Ratio, SII and RII were used for the economic status and education dimensions of inequality,. For the place of residence (urban, rural) and sex (male, female), only the Difference and Ratio were calculated. The reason being, the RII and SII require equity stratifers that are ordered, and wealth and education are such dimensions of inequality. Difference was calculated as health seeking behavior for childhood pneumonia in economic status (the richest group minus the poorest group) as well as education (secondary or higher education minus the uneducated group). Similarly, for the place of residence, Difference pertains to that between urban and rural populations. Ratio (R) is calculated as R = Y_high_ / Y_low_. For place of residents, Y_high_ is urban and Y_low_ is rural residents; for educational status, Y_high_ refers to the most advantaged subgroups with secondary school education or higher and Y_low_ refers to the most disadvantaged subgroups with no education. Ratio for economic status represents the richest quintile relative to the poorest quintile while that for sex, it is the ratio of health seeking behavior for female to male childhood pneumonia treatment.

SII and RII use a generalized linear model with a logit link for the two ordered measures with ranking of weighted samples in order from most disadvantaged (poorest and uneducated - ranked 0) to the most advantaged (at least secondary education or higher and the richest subgroups - ranked 1). Health seeking behavior for childhood pneumonia treatment was predicted for the two extremes and the difference in the predicted value between rank 1 and rank 0 produced SII. The RII was determined by dividing the predicted health seeking behavior for childhood pneumonia treatment for rank 1 by that of rank 0, covering the entire distribution.

Ninety-five percent (95%) Uncertainty Intervals (UI) were computed around point estimates to measure statistical significance. For statistical significance and to show inequality, the lower and upper bounds of the UI do not include zero (0) for Difference and SII. For R and RII, inequality exists if UIs do not include one (1). To interpret that there was inequality across the years, UIs of the summary measures for each survey year should not overlap. To maintain quality of the scientific presentation of the evidence contained in the paper, this study followed Strengthening the Reporting of Observational Studies in Epidemiology (STROBE) guidelines [25].

### Ethical consideration

The analysis was completed using data which was publicly available through the demographic and health surveys in the WHO HEAT software. Organizations which commissioned, funded and managed the surveys completed the necessary ethical procedures. ICF international and the Institutional Review Board (IRB) in Ethiopia approved the DHS surveys, ensuring necessary protocols were in compliance with the U.S. Department of Health and Human Services regulations for the protection of human subjects.

## Results

### Trends of health seeking behavior across inequality dimensions

The total sample included 2, 284 children in the three rounds of the survey (2005, 2011, and 2016). Approximately half (*n* = 1162, 50.8%) were male, the majority lived in rural regions (*n* = 2132; 93.3%) and 72.7% (*n* = 1661) had mothers with no education. In terms of economic status, health seeking behavior level increased for the richest group between 2005 (35.2%) and 2011 (61.7%) and then decreased to 37.6% in 2016. Whereas in the poorest quintile, there was a decrease from 25.8% in 2005 to 15.4% in 2011 followed by increase to 24.9% in 2016. Health seeking behavior level was higher among urban than rural residents – the difference remained constant across all three rounds of the survey. Table 1 illustrates health seeking behaviour level for pneumonia treatment in urban (43.0%, 46.8% and 55.7%) and rural region (21.7%, 25.0%, and 27.4%) in 2005, 2011 and 2016, respectively. Overall, the percentage of under-five children with suspected pneumonia taken to health facilities for treatment was 22.6%, 27.0% and 29.4% in 2005, 2011 and 2016, respectively.

**Table 1:** Trends of health seeking behavior in under-five children with symptoms of pneumonia in Ethiopia (EDHS 2005–2016)

Dimension of inequalities	Year					
	2005		2011		2016	
	% (95% UI)	Popn	% (95% UI)	Popn	% (95% UI)	Popn
Economic status						
Quintile 1 (poorest)	25.86 (15.94,39.09)	157	15.48 (9.77,23.64)	188	24.96 (13.12, 42.29)	132
Quintile 2	14.21 (7.30,25.86)	173	25.24 (16.24, 37.01)	148	25.65 (16.84, 37.02)	172
Quintile 3	24.98 (16.66,35.69)	219	22.06 (14.65, 31.81)	196	27.40 (19.96, 36.37)	175
Quintile 4	17.04 (11.01, 25.43)	163	33.17 (21.95, 46.69)	172	36.84 (24.87, 50.68)	147
Quintile 5 (richest)	35.21 (23.08, 49.61)	110	61.72 (45.06, 76.02)	67	37.62 (21.64, 56.85)	62
Education						
No education	18.01 (13.64,23.39)	636	24.61 (19.35, 30.75)	550	26.09 (20.10, 33.14)	475
Primary school	35.30 (25.32,46.76)	165	27.67 (19.45, 37.75)	199	35.21 (26.59, 44.90)	177
Secondary school +	NA	20	NA	22	44.45 (21.85, 69.61)	37
Residence						
Rural	21.74 (17.47, 26.71)	786	25.04 (20.34, 30.42)	703	27.48 (22.30, 33.36)	643
Urban	43.04 (24.97, 63.17)	36	46.88 (24.01, 71.15)	69	55.74 (35.44, 74.29)	47
Sex						
Female	22.41 (16.63, 29.48)	401	28.70 (22.13, 36.32)	380	26.71 (20.40, 34.14)	341
Male	22.95 (17.65, 29.27)	421	25.35 (19.08, 32.85)	392	32.10 (24.94, 40.20)	349
Overall	22.60		27.00		29.40	

### Level and trends of inequalities in health seeking behavior for childhood pneumonia treatment

Table 2 shows there was no inequality based on economic status in 2005. In 2011, however, all measures of absolute (D, SII) and relative (R, RII) economic inequality showed a significant difference between the groups. In 2016, the simple measures (D, R) did not indicate economic inequality, but the complex measures (SII, RII) showed a slight significant difference - the richest families were more likely to seek treatment for pneumonia than the poorest families. Interestingly, the pattern over time showed a peak in inequality in health seeking behavior level in 2011 across all the measures; it increased from 2005 to 2011 and then decreased again 5 years later in 2016.

**Table 2:** Inequalities in under-five children with symptoms of pneumonia who taken into appropriate health facilities in Ethiopia (EDHS 2005–2016)

Dimension of Inequalities	Measures of inequalities	Years		
		2005	2011	2016
		% (95%UI)	% (95%UI)	% (95%UI)
Economic status	D	9.35 (−8.28, 26.98)	46.24 (28.92, 63.56)	12.66 (−10.76, 36.09)
	R	1.36 (0.56, 2.15)	3.98 (1.94, 6.02)	1.50 (0.35, 2.65)
	RII	1.36 (0.75, 1.96)	4.04 (2.25, 5.84)	1.77 (1.03, 2.51)
	SII	7.02 (−3.13, 17.18)	35.61 (25.31, 45.92)	16.67 (4.81, 28.53)
Education	D	NA	NA	18.35 (−8.26, 44.98)
	R	NA	NA	1.70 (0.62, 2.78)
	RII	NA	NA	2.07 (1.08, 3.06)
	SII	NA	NA	21.14 (7.59, 34.68)
Residence	D	21.30 (0.98, 41.61)	21.84 (−4.13, 47.81)	28.26 (7.14, 49.37)
	R	1.97 (0.97, 2.98)	1.87 (0.78, 2.95)	2.02 (1.18, 2.87)
Sex	D	−0.54 (−9.09, 8.00)	3.35 (−6.53, 13.23)	−5.38 (−15.64, 4.87)
	R	0.97 (0.60, 1.34)	1.13 (0.71, 1.54)	0.83 (0.54, 1.12)

Inequality in health seeking behavior based on education was only available for 2016. According to absolute and relative complex measures of SII and RII, inequality was present in 2016 when the more educated households were more likely to seek treatment for a child suspected of pneumonia than households with uneducated mothers. Neither absolute nor relative simple measures (D, R) indicated educational inequality. Since the value of the outcome variable is missing for one or more categories of education, it was not possible to calculate educational inequality in 2005 and 2011.

The results indicated absolute (D) residence inequality in 2005, absence of any type of inequality in 2011 and both absolute (D) and relative (R) place of residence inequality in 2016 (Table 2). In the last survey (2016), families in urban dwellings were more likely to seek treatment for pneumonia than those in rural dwellings. None of the inequality measures indicated sex inequality in health seeking behavior in under-five children in Ethiopia between 2005 and 2016.

## Discussion

Using the WHO health equity monitor database, this study provided the magnitude and time-trend analysis of socio-economic, sex and area-based inequalities in under five children with symptoms of pneumonia taken to a health facility in Ethiopia. Our findings show the percentage of under-five children with pneumonia symptoms taken to health facility was 22.6%, 27.0% and 29.4% in 2005, 2011 and 2016, respectively. This is inconsistent with a previous study of sub-Saharan African countries, where care seeking behavior by caregivers of children under-five years of age with suspected pneumonia was far higher in Ethiopia (86%) [26]. The WHO states that correct care includes properly diagnosing and treating pneumonia [27]. Recent studies show less than 60% of children under-five with symptoms of pneumonia, worldwide, are taken to an appropriate provider; in the least-developed countries, this coverage is under 50% [27]. The overall results of our work demonstrate existence of inequalities in health seeking behavior favoring households with higher wealth and more education living in urban regions with no significant sex differential in all the survey periods.

Generally, all measures of economic inequality (i.e., Difference, Ratio, SII and RII) were significantly higher in 2011, and only continued to 2016 with complex measures of SII and RII. Similar to this study’s findings, care seeking for pneumonia among wealthier quintiles has also been shown in other SSA countries [8, 26, 27]. The study also revealed existence of education-based disparity in under-five children with pneumonia symptoms who were taken to a health facility - more educated sub-groups had higher access to treatment in 2016. Available evidence supports the finding that higher maternal education increases the likelihood of receipt of health care services for pneumonia [26, 27].

Although residence related inequality existed in 2005 and 2016 using the Difference measure, this urban – rural differential did not significantly increase from 2005 to 2016, as seen by overlapping UIs. In 2016, inequality was evident since twice as many urban residents sought health treatment for pneumonia than rural residents. To date, there have been mixed findings regarding rural-urban residence; some studies indicate they are more disadvantaged because of poverty and little access to health services [27] while others have not shown a significant association between rural-urban residence and care seeking [26]. Timely recognition of symptoms of pneumonia is the first step to seeking interventions, however, studies have indicated this does not generally take place in underserved settings around the world, such as rural or urban slums [28]. Therefore, there is a need to increase awareness in communities about the importance of early recognition and subsequent help seeking of appropriate services.

Interestingly, we found no significant male-female difference observed in help seeking services for pneumonia. Absence of gender disparity around important child health care services is crucially necessary as gender disparity has been greatly linked with child survival, especially in LMICs [29]. Although it is encouraging to find no sex inequality in seeking services for pneumonia in Ethiopia, it remains important to remove the existing disparities around the other dimensions of inequality.

The investigation of health care inequalities through different measures and dimensions of inequality presents disparities from wider and sometimes conflicting perspectives; a single summary measure of inequality is not enough to understand what is truly happening in terms inequalities [24]. The simple summary measures (i.e., Difference and Ratio) are restricted to two extreme groups of a sub-population in an equity stratifer and do not include the sub-groups in the middle. This could lead to a biased conclusion, particularly when there is a population shift in a subpopulation of interest over time [24].

Our paper reveals important policy implications for the community, policy makers, and concerned stakeholders. Socioeconomic and residence inequalities in health seeking behavior in childhood pneumonia persisted over the 11 years. This issue must be appropriately addressed, and interventions accordingly designed to assist the vulnerable portion of the population with lower health seeking behaviors. We suggest focusing on promotion strategies, with a holistic approach, to address health seeking behavior in childhood pneumonia inequality. By reducing inequities, increasing promotion and strengthening prevention, the country can work towards the SDGs.

The WHO and UNICEF have signaled the ongoing challenges of differentiating pneumonia from other problems with similar clinical presentation. In their 2016 report entitled “One is too many, ending child death from pneumonia and diarrhoea”, UNICEF called for better diagnostics to detect pneumonia [8]. The report highlighted the need to train health care professionals in Integrated Management of Childhood Illness (IMCI) and integrated Community based Case Management (iCCM) to better diagnose and treat multiple conditions of pneumonia [8]. While local governments work towards detecting all cases of pneumonia, special attention must be directed towards the most affected subpopulations. Scaling up of diagnosis and appropriate coverage of treatment for children with pneumonia should be considered at the national level to ensure the most disadvantaged children are reached. Countries are aiming for 80% coverage of appropriate pneumonia case management in small scale divisions (i.e., districts) by the end of 2025 [9]. However, at a national coverage of 29%, Ethiopia is far from the goal. More work is needed in terms of diagnosis and management with more attention drawn to children born to women residing in rural areas as well as women who are in the lower quintiles (poor/poorest) and less educated. To reach the global targets for child health, and ultimately meet the universal health coverage goal for child health service (including interventions for pneumonia), nations should adopt the research and intervention protocols of the WHO to identify and treat pneumonia cases in a timely fashion.

Mulholland et al. (2008) stated that the key strategies for the prevention of childhood pneumonia include case management and immunization with the newer vaccines against *Haemophilus influenzae* type b (Hib) and pneumococcus [30]. With increased efforts to introduce the interventions into communities, there will be a tendency to exacerbate the existing inequity in access, with the already disadvantaged continuing to receive little of the services. Therefore, work is required to increase the coverage of the interventions among the disadvantaged groups while also maintaining coverage for the advantaged groups [30].

In addition to working on a targeted health education and promotion approach, eliminating the observed inequality in the utilization of treatment for pneumonia requires government level policy that addresses issues of inequity within the health care structure. In Ethiopia, disparity in access to interventions, including those for pneumonia, remain a major problem and the government is working towards overcoming the structural inequality through increasing coverage of both health facilities and skilled health personnel to enhance the system’s capacity for diagnosis and management [31].

The study had several strengths. First, the inequality analysis was based on the established WHO health equity monitor database which provided quality data for the results in this paper. We used the 2019 update of the database, so it captured the current status of under-five children with pneumonia symptoms taken to a health facility from information obtained through the latest three rounds of the Ethiopia DHS. Second, by using different inequality summary measures, we investigated the nature of pneumonia related health seeking behavior inequality from several perspectives.

The study has limitations. It used nationally representative EDHS data which could not be generalized to areas below the sub-national regions. Countries around the world are aiming for 80% coverage of appropriate pneumonia case management in small scale divisions by the end of 2025, as set out by the GAPPD [9]. As discussed, Ethiopia’s national coverage of 29% is far from the stated goal. We recommend spatial analysis of the service that include small geographical areas to see how they are performing in relation to the global goal. We also suggest qualitative work involving decision makers to better understand the problems underpinning inequality in seeking pneumonia treatment from the health system structure’s standpoint. The WHO equity monitor database does not disaggregate age for pneumonia help seeking inequality; age should have been used as an equity stratifier to know the specific age bracket treatment for pneumonia. Finally, the study did not decompose the observed inequality in under-five children with pneumonia symptoms taken to a health facility to determinants or factors that could help explain the inequalities observed.

## Conclusions

The study showed both socioeconomic and area-based inequalities in health seeking behavior of childhood pneumonia disfavoring children in the lower socioeconomic status and households residing in rural areas in Ethiopia. Inequality in under-five children with pneumonia symptoms taken to health facility was observed in three of the survey years and across economic status, educational status and place of residence between the 2005 and 2016 EDHS. The inequality remained constant in place of residence and narrowed and /or decreased in economic status inequality. Interestingly, we did not observe sex related inequality in all the survey periods.

Government and key stakeholders (i.e., health professionals, educators, media) can partner to create awareness about and advocate for the importance of early diagnosis and treatment of childhood pneumonia and health seeking practices. This is important for the general public and lower socio-economic subgroups and rural residents identified at a disadvantage for help seeking behaviour. The country’s health system capacity for diagnosis and management should be further examined. Future studies should also investigate the potential determinants related to the observed inequality in health seeking behavior of childhood pneumonia.

## Data Availability

The datasets generated and/or analyzed during the current study are available in the WHO’s HEAT version 3.1 [https://www.who.int/gho/health_equity/assessment_toolkit/en/].

## Abbreviations

UI: Uncertainty Interval
D: Difference
DHS: Demographic and health survey
EA: Enumeration area
EDHS: Ethiopia Demographic and Health Survey
HEAT: Health Equity Assessment Toolkit
R: Ratio
RII: Relative Index of Inequality
SDG: Sustainable development goal
SII: Slope Index of Inequality
WHO: World Health Organization
